# The EF‐1α promoter maintains high‐level transgene expression from episomal vectors in transfected CHO‐K1 cells

**DOI:** 10.1111/jcmm.13216

**Published:** 2017-05-30

**Authors:** Xiaoyin Wang, Zhongjie Xu, Zhengwei Tian, Xi Zhang, Danhua Xu, Qin Li, Junhe Zhang, Tianyun Wang

**Affiliations:** ^1^ Department of Biochemistry and Molecular Biology Xinxiang Medical University Xinxiang Henan China; ^2^ Henan Collaborative Innovation Center of Molecular Diagnosis and Laboratory Medicine Xinxiang Henan China; ^3^ Life Science and Technology Xinxiang Medical University Xinxiang Henan China; ^4^ Test Laboratory Xinxiang Medical University Xinxiang Henan China

**Keywords:** episomal vectors, transgene expression, regulatory element, transgene stability

## Abstract

In our previous study, we demonstrated that episomal vectors based on the characteristic sequence of matrix attachment regions (MARs) and containing the cytomegalovirus (CMV) promoter allow transgenes to be maintained episomally in Chinese hamster ovary (CHO) cells. However, the transgene expression was unstable and the number of copies was low. In this study, we focused on enhancers, various promoters and promoter variants that could improve the transgene expression stability, expression magnitude (level) and the copy number of a MAR‐based episomal vector in CHO‐K1 cells. In comparison with the CMV promoter, the eukaryotic translation elongation factor 1 α (EF‐1α, gene symbol *EEF1A1*) promoter increased the transfection efficiency, the transgene expression, the proportion of expression‐positive clones and the copy number of the episomal vector in long‐term culture. By contrast, no significant positive effects were observed with an enhancer, CMV promoter variants or CAG promoter in the episomal vector in long‐term culture. Moreover, the high‐expression clones harbouring the EF‐1α promoter tended to be more stable in long‐term culture, even in the absence of selection pressure. According to these findings, we concluded that the EF‐1α promoter is a potent regulatory sequence for episomal vectors because it maintains high transgene expression, transgene stability and copy number. These results provide valuable information on improvement of transgene stability and the copy number of episomal vectors.

## Introduction

Expression vectors play important roles in gene therapy. Vectors currently used for gene expression have a number of limitations 1, 2, 3, such as random integration into the host genome or only transient retention. Random integration may lead to insertional mutagenesis and silencing of the transgene. Therefore, the ideal vector, especially for gene therapy, should be retained in cells without integration. Nonviral episomal plasmid vector pEPI was first developed by Piechaczek *et al*. 4. pEPI requires a scaffold/matrix attachment region (S/MAR) linked to an expression unit 5, 6. Active transcription upstream of the S/MAR running into this sequence is required and is probably sufficient for episomal replication. When S/MAR is placed upstream of a transcription termination site or is deleted, the vector is lost or gets integrated 7. The pEPI vector replicates independently at a low copy number in all verified cells. In addition to the low copy number, other drawbacks of the pEPI vector include unstable or low transgene expression 8, 9. In the subsequent studies, considerable efforts were made to improve pEPI‐based vectors, such as insertion of regulatory *cis*‐acting elements and reducing the amount of bacterial sequence 10, 11.

In our previous study, we constructed an episomal vector harbouring a 387‐bp DNA sequence comprising a characteristic MAR motif and evaluated different promoters in the episomal vectors. We found that the CMV promoter performed the best in terms of expression magnitude and stability 11, 12. However, expression of the transgene was unstable, the copy number was low, and the expression magnitude was still unsatisfactory.

Composition of a plasmid vector, including promoters, enhancer, polyadenylation signals and other expression elements, influences transgene expression magnitude and stability. Although the CMV promoter provides high gene expression, there are reports that with the extended cultivation periods, productivity decreases 13, 14. CMV is prone to transcriptional silencing that is associated with DNA methylation 15, 16. By contrast, several strong promoters have been exploited with the aim of long‐term, potent expression of a gene. Promoters of mammalian origin have been used most widely for this purpose, among which the human eukaryotic translation elongation factor 1 alpha (EF‐1α, gene symbol *EEF1A1*) promoter is constitutively active in a broad range of cell types. The EF‐1α promoter is often active in cells in which viral promoters fail to express the controlled genes and in cells in which the viral promoters are gradually silenced 17, 18, 19. Some studies have indicated that promoters of endogenous mammalian genes like *EEF1A1* may be more resistant to silencing than viral promoters are 20, 21, 22. The EF‐1α promoter used in conjunction with flanking regions of the CHO EF‐1α gene is more active in CHO cells as compared with the use of CMV and SV40 promoters alone 23, 24.

To further enhance the function of promoters, various enhancer elements have been added upstream of the promoters. In this study, we evaluated performance of various enhancer elements, the EF‐1α promoter, CAG promoter (a fusion promoter comprising a CMV enhancer, the chicken β‐actin promoter and a rabbit β‐globin splice acceptor site) and CMV promoter mutants in the episomal vector and tested their transgene expression level and stability in CHO‐K1 cells. Our findings should benefit researchers designing episomal vectors to achieve high expression and long‐term stability.

## Materials and methods

### Construction of the vectors

Based on previously described vector pEM (Fig. S1A), the CMV promoter mutants (inclusion of cytosines at positions 404 and 542 that were point‐mutated to guanosines, Fig. S1B) and three different enhancer elements added upstream of the promoters (Fig. S1C) were synthesized chemically by Sangon Biotech Co., Ltd. (Shanghai, China). The promoter and enhancer sequences are listed in Figure S2. The EF‐1α and CAG promoters (Fig. S1D) were generated by polymerase chain reaction (PCR). To achieve directional cloning, *Ase* I and *Nhe* I restriction sites were introduced at the 5′ ends of the primers. The PCR cycling conditions were as follows: four cycles of 95°C for 3 min., 60–56°C for 30 sec., 72°C for 40 sec.; followed by 20 cycles at 94°C for 40 sec., 55°C, 72°C for 40 sec.; and a final extension step at 72°C for 3 min. The PCR products were recovered and their sequences were confirmed, followed by digestion with *Ase* I and *Nhe* I (Takara Biotechnology Co., Ltd., Dalian, China). The products were then ligated into the pEM vector to obtain vectors containing the EF‐1α or CAG promoter (Fig. S1D and E). The schematics of the constructs are shown in Figure S1.

### Cell culture and transfection

Chinese hamster ovary K1 cells (CHO‐K1) (provided by the Institute of Laboratory Animal Sciences, Beijing, China) were cultured in Dulbecco's modified Eagle's medium (Gibco, Carlsbad, CA, USA) supplemented with 10% of foetal bovine serum (Gibco, Grand Island, NY, USA) and 1% of a penicillin–streptomycin solution (Solarbio, Beijing, China) in a humidified incubator at 37°C and 5% CO_2_. The cells were cultured at a density of 8 × 10^4^/well in 12‐well plates. After the cells reached 80–90% confluence, triplicate transfections were performed for each vector using Lipofectamine^®^ 2000 (Invitrogen, Waltham, MA, USA) or PolyJet™ (SignaGen Laboratories, Rockville, MD, USA), according to the manufacturer's instructions. Stably transfected cells were selected 48 hrs after transfection by the addition of Geneticin (G418; Invitrogen) to the medium at a concentration of 500 μg/ml with selection for 2 weeks. The selected cells were subcultured into 96‐well plates to obtain monoclonal clones using the limiting dilution method. Stable clones were then cultured in either the presence or absence of G418 (250 μg/ml).

### Flow cytometry

CHO‐K1 cells were obtained by screening at different time‐points after the transfection. eGFP gene expression was examined under an Olympus IX71 fluorescence microscope (Olympus, Tokyo, Japan). Microscope settings were as follows: applicable mirror unite U‐MN, excitation maximum 488, emission maximum 507 and exposure time 100 ms. The percentage of enhanced green fluorescent protein (eGFP)‐expressing cells (48 hrs post‐transfection) and eGFP mean fluorescence intensity (MFI) of each sample were analysed using a FACSCalibur cytometer (Becton Dickinson, Franklin Lakes, NJ, USA). FACS settings were as follows: FSC voltage 60, SCC voltage 300 and FITC voltage 380. The percentage of eGFP‐expressing cells was determined by eGFP antibody labelling. Briefly, CHO‐K1 cells were digested with trypsin and collected, followed by resuspending in 100 μl of a mouse anti‐GFP antibody solution (ZSGB‐Bio, Beijing, China), and finally, the cells were analysed on the flow cytometer. The numbers of eGFP‐expressing and eGFP‐non‐expressing cells were calculated according to the flow cytometer results, and the transfection efficiency was calculated as the ratio of the number of eGFP‐expressing cells to the total cell number.

### Stability testing

Cells were passaged in 6‐well plates and cultured further. The MFI for each vector type was measured using the FACSCalibur cytometer, and the retention of eGFP expression for each vector was calculated as the ratio of the MFI values at the end to those at the start of stability testing. The retention rate was calculated in accordance with the eGFP protein levels with or without G418 in CHO‐K1 cells after 30 generations, as compared with the original eGFP expression levels. The retention rate is defined as MFI of the original monoclonal cells/MFI of monoclonal cells with or without G418 after 30 generations. All the experiments were repeated three times.

### Plasmid rescue experiments

A modified Hirt protocol 11, 25 was used to isolate extrachromosomal DNA from CHO‐K1 monoclonal cells transfected with the pEMEα vector. *Escherichia coli* (Sangon Co., Ltd., Shanghai, China) was electroporated with the Hirt extract from approximately 10^6^ stably transfected cells. *E. coli* transformants were selected using agar plates containing 100 μg/ml kanamycin. Plasmid DNA was prepared from individual resistant clones, digested with *Nhe* I or *Nhe* I/*Ase* I and visualized on 1.0% agarose gels, with the aim of verifying the episomal status of pEMEα vectors in CHO‐K1 cells.

### Southern blotting

For this analysis, genomic DNA and extrachromosomal DNA plasmid (from a Hirt extract involving 10^7^ CHO‐K1 cells transfected with pEMEα) were isolated, digested with the single‐cutting restriction enzyme *Ase* I, separated on 0.7% agarose gels (20 V, 20 mA overnight) and blotted onto Amersham Hybond‐N+ paper, according to the manufacturer's instruction (GE Healthcare, Buckinghamshire, UK). Finally, an eGFP probe was labelled with Digoxin (Roche, Mannheim, Germany). The hybridization was performed in Church buffer (0.25 M sodium phosphate buffer pH 7.2, 1 mM EDTA, 1% of BSA and 7% of SDS) at 65°C for 16 hrs.

### Fluorescence *in situ* hybridization (FISH)

Fluorescence *in situ* hybridization was performed to determine the episomal sites and gene copy number of the pEMEα vector. Monoclonal cells transfected with pEMEα and showing different expression levels were collected. eGFP served as a probe and was labelled using a Digoxigenin‐Nick translation kit (Roche, Mannheim, Germany). Samples were counterstained with 1 μg/ml 4′,6′‐diamidino‐2‐phenylindole before examination under a Leica DMRB fluorescence microscope with a Leica DC 300 f camera. Approximately 50 visual fields were examined, and mean copy numbers were calculated. Fifty metaphase plates were analysed by FISH for each vector type.

### Quantitative PCR

Episomal DNA was extracted from the cells as described for the plasmid isolation assay. The following primers were used in the subsequent PCR analysis: 5′‐GATGGGGTACCCTTCATCC‐3′ (glyceraldehyde‐6‐phosphate dehydrogenase [*G6PDH*] forward) and 5′‐GCTCTGACTCCTCAGGGTTG‐3′ (*G6PDH* reverse); and 5′‐GCTGGTTTAGTGAACCGTCAG‐3′ (eGFP forward) and 5′‐AGGTGGCATCGCCCTCGCCC‐3′ (eGFP reverse). Ready‐to‐use “hot‐start” FastStart DNA Master PLUS SYBER Green I fluorescent reaction mix (Roche, Mannheim, Germany) and an ABI 7500 SYBER Fluorescence quantitative PCR instrument (Applied Biosystems, Foster City, CA, USA) were used for PCRs, which were run for 40 cycles using the manufacturer‐recommended parameters. Relative eGFP copy numbers were calculated by the 2^−ΔΔCt^ method. All the experiments were repeated three times.

### Bioinformatics analysis

Specific transcription factor‐binding sites were identified by means of the MatInspector software (http://www.genomatix.de/products/index.html) 26.

### Statistical analysis

All experimental data were analysed in the SPSS 18.0 software (SPSS Inc., Chicago, IL, USA). Data are reported as mean ± standard deviation (S.D.). A post‐analysis of variance, multiple comparison procedure, was performed next to assess pairwise differences in expression confirmed by analysis of variance. Differences with *P* values <0.05 were considered statistically significant.

## Results

### Transfection efficiency and transient expression

**Figure 1:**
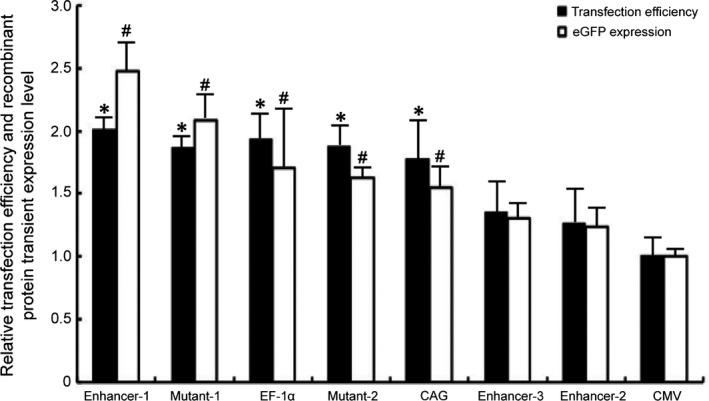
Positive recombinant protein expression rates and relative eGFP expression levels in transfected CHO‐K1 cells. All the experiments were repeated three times. The number of cell expressing the eGFP gene and eGFP expression in CHO‐K1 cells was analysed by flow cytometry 48 hrs post‐transfection.*^,#^, Indicates that transfection efficiency and eGFP expression levels from Enhancer‐1, Mutant‐1, EF‐1α, Mutant‐2 and CAG were significantly higher than those from CMV, (Student's *t*‐test, *P* < 0.05).

The constructed plasmids were transfected into CHO‐K1 cells, and for each plasmid, the number of cells expressing the eGFP gene and eGFP protein levels (MFI) was analysed by flow cytometry at 48 hrs post‐transfection. The proportion of cells expressing the recombinant protein was the highest for the plasmid with Enhancer‐1 (84.3%), followed by Mutant‐1 (82.2%), the EF‐1α promoter (80.9%), Mutant‐2 (78.6%), the CAG promoter (74.5%), Enhancer‐3 (57.1%), Enhancer‐2 (52.4%) and the CMV promoter (42.3%) in CHO‐K1 cells. When the transfection efficiency for plasmid pEM (including the CMV promoter) was set to 1.0, the Enhancer‐1, Mutant‐1, EF‐1α, Mutant‐2, CAG, Enhancer‐3 and Enhancer‐2 values were 2.01, 1.86, 1.93, 1.87, 1.77, 1.35 and 1.24, respectively (Fig. 1). The results indicated that in comparison with the CMV promoter, except for Enhancer‐3 and Enhancer‐2, all other elements enhanced the recombinant protein expression (*P* < 0.05). The positive effect on eGFP expression exerted by these vectors correlated with the corresponding transfection efficiency (Fig. 1). The eGFP protein amounts were the highest for the vector containing Enhancer‐1, followed by Mutant‐1, EF‐1α, Mutant‐2, CAG, Enhancer‐3, Enhancer‐2 and CMV (Fig. 1). When the eGFP protein quantity from the CMV promoter‐carrying vector was set to 1.0, the eGFP levels in Enhancer‐1, Mutant‐1, EF‐1α, Mutant‐2, CAG, Enhancer‐3 and Enhancer‐2 were 2.48, 2.08, 1.71, 1.63, 1.55, 1.30 and 1.24, respectively. Thus, the protein levels yielded by Enhancer‐1, Mutant‐1, EF‐1α, Mutant‐2 and CAG were significantly higher than those afforded by the CMV promoter (*P* < 0.05).

### Stability of expression of the transgene

**Figure 2:**
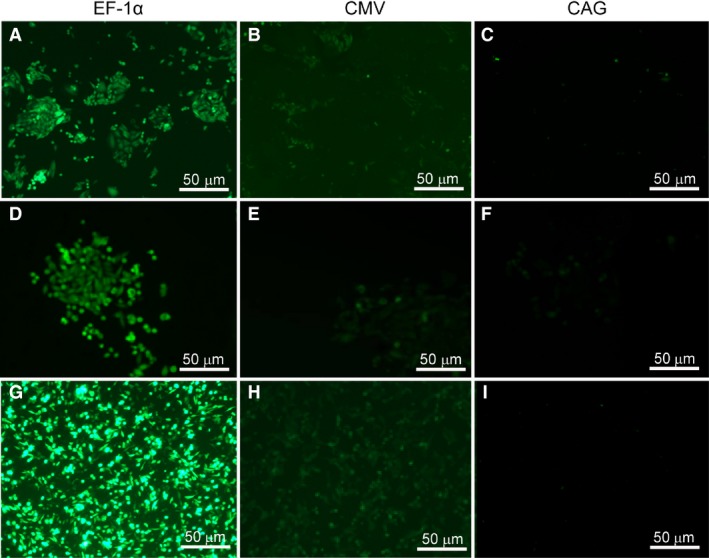
Number of clones and eGFP expression in transfected CHO‐K1 cells. (**A**–**C**) represent number of clones in CHO‐K1 cells transfected the plasmid with EF‐1α, CMV and CAG promoter, respectively. The presence of EF‐1α promoter exhibited many clones, CMV exhibited few clones, and CAG promoter exhibited no clones. (**D**–**F)** represent positive clones in CHO‐K1 cells transfected the plasmid with EF‐1α, CMV and CAG promoter, respectively. (**G**–**I)** represented eGFP expression at 2 weeks post‐transfection in CHO‐K1 cells transfected the plasmid with EF‐1α, CMV and CAG promoter, respectively.

At 48 hrs after transfection, stably transfected cells were selected by the addition of Geneticin (G418) to the medium at a concentration of 500 μg/ml with selection for 2 weeks. During this selection process, the differences between various vectors were reflected in the number of clones and eGFP expression magnitude (Fig. 2). The EF‐1α promoter yielded the most number of clones and strongest eGFP expression as compared with all the other regulatory elements (Fig. 2A, D, G). After 2 weeks, eGFP expression in CHO‐K1 cells transfected with the plasmid carrying the EF‐1α promoter was significantly higher than that in the cells transfected with the plasmid containing the CMV promoter (Fig. 2B, E, H), and little eGFP expression was observed in cells harbouring each of the other six vectors (a representative example is the plasmid containing the CAG promoter, Fig. 2C, F, I).

### Evaluation of the EF‐1α promoter in stably transfected CHO‐K1 cells

**Figure 3:**
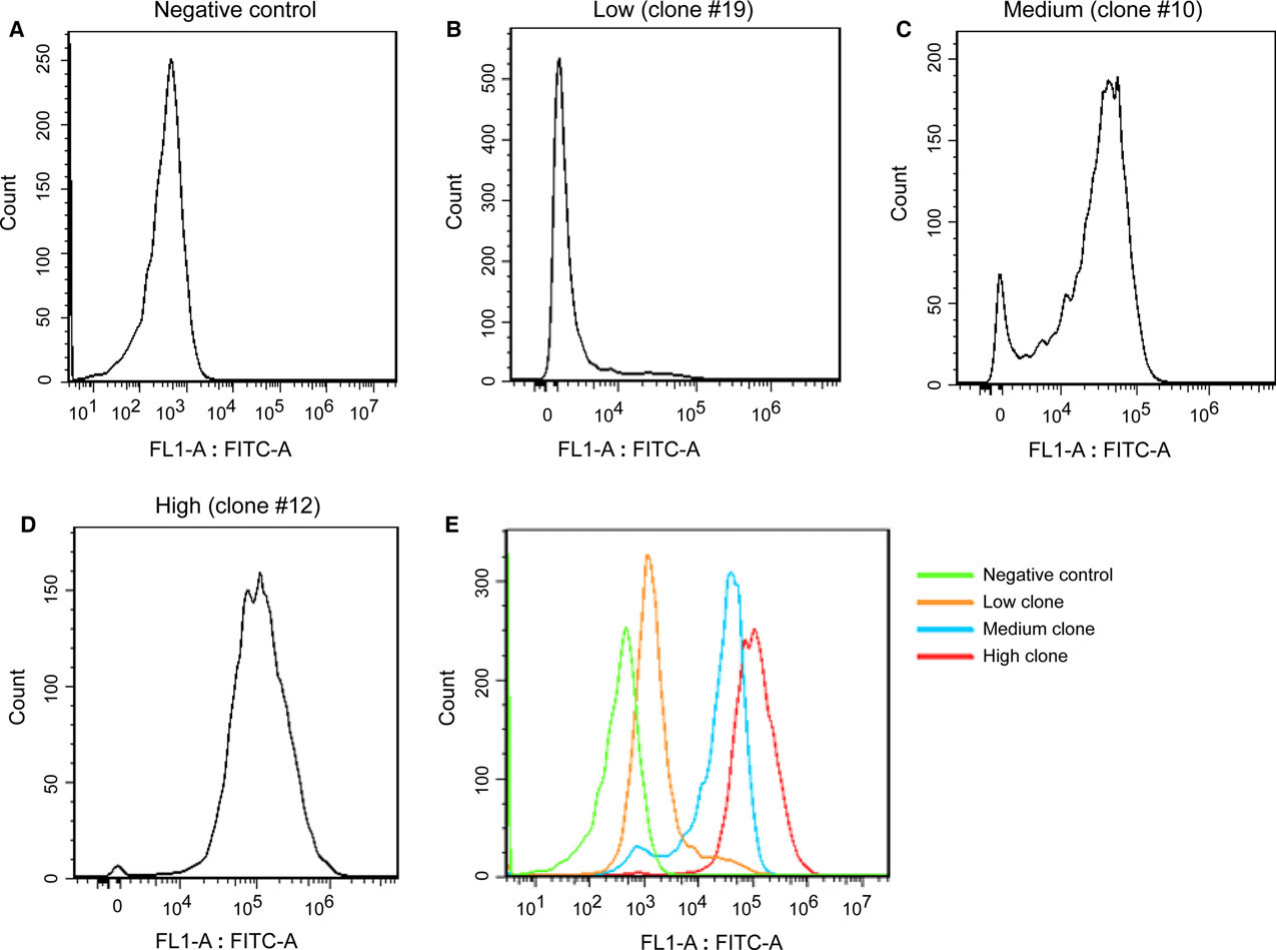
eGFP expression level from isolated pEMEα clones. Monoclonal cells were established in CHO‐K1 cells using the limiting dilution method. Clones were grown to 60–70% confluence and subjected to FACS analysis. The clones can be divided into three groups based on their eGFP expression level. (**A**) Negative control; (**B**) group with high expression represented by eight clones (*e.g*. clone #12); (**C**) group with medium expression represented by six clones (*e.g*. clone #10); (**D**) group with low expression represented by eight clones (*e.g*. clone #19); (**E**) eGFP expression level in high, medium and low clones.

Given that the EF‐1α promoter performed best in terms of expression levels and clone numbers, we chose CHO‐K1 cells transfected with the EF‐1α promoter‐carrying vector (pEMEα) for our subsequent experiments. The cells were subcultured into 96‐well plates to obtain monoclonal cultures, and FACS analysis of CHO‐K1 clones carrying pEMEα revealed uniform expression levels (Fig. 3). We selected three groups of monoclonal cells: group A showed high expression (the eGFP expression level was 1.35 × 10^5^ to 2.30 × 10^5^ MFI, *e.g*. clone #12, Fig. 3B); group B showed medium expression (the eGFP expression level was 3.53 × 10^4^ to 4.86 × 10^4^, *e.g*. clone #10, Fig. 3C); and group C had low expression (the eGFP expression level was 4865‐10002, *e.g*. clone #19, Fig. 3D). Compared with the negative control, the average difference in eGFP expression for high‐, medium‐ and low‐expression clones was 358.3‐, 82.6‐ and 16.5‐fold, respectively. The average eGFP level in the high‐expression clone was 4.8, 21.1‐fold higher than that in the medium‐expression and the low‐expression clones, respectively (Fig. 3E).

### Plasmid rescue experiments

**Figure 4:**
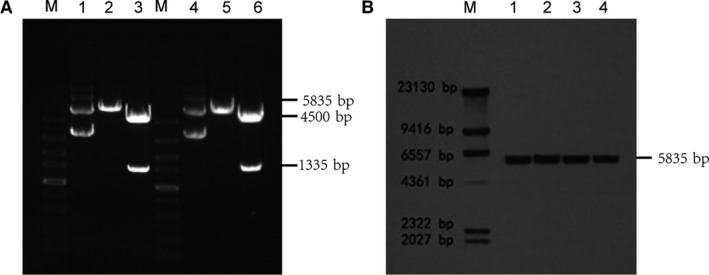
Analysis of episomal *versus* integrated status of pEMEα. (**A**) Rescue experiments in *E. coli* using Hirt extract from CHO‐K1 cells transfected with pEMEα. Hirt DNA was isolated and digested with *Nhe* I or Nhe I/*Ase* I. In monoclonal #12,10,19 cells, the length of plasmid DNA from CHO‐K1 cells was found to be identical to the original vector DNA(one example is shown). M, DL5000 Marker; Lane 1, pEMEα plasmid; Lane 2, pEMEα plasmid digested with *Nhe* I; Lane 3, pEMEα plasmid digested with *Nhe* I/*Ase* I; Lane 4, Hirt DNA isolated from a single bacterial clone; Lane 5, Hirt DNA isolated from a single bacterial clone digested with *Nhe* I; Lane 6, Hirt DNA isolated from a single bacterial clone digested with *Nhe* I/Ase I. (**B**)Southern analysis of DNA isolated from CHO‐K1 cells transfected with pEMEα. Hybridization was done as described in Methods. The hybridization pattern of one representative clone is shown for each construct. Lanes, M: DNA Marker; 1: plasmid DNA as a control linearized by digested with *Nhe* I; 2,3,4: Hirt plasmid extract from monoclonal #12,10,19 cells digested with *Nhe* I, respectively.

Using the Hirt DNA extraction method, we extracted extrachromosomal DNA from cells of clone #12, 10 and 19 carrying pEMEα, and the isolated plasmids were identified by digestion with the restriction enzymes. DNA was isolated from 10 clone using the Hirt extraction method, transformed into *E. coli* and subjected to digestion with *Nhe* I or *Nhe* I /*Ase* I. For all three clones, the length of the plasmid DNA from CHO‐K1 cells was found to be identical to the original vector DNA (one example is shown in Fig. 4A). *Ase I* /*Nhe I* digestion was expected to yield 1335‐bp and 4500‐bp fragments, whereas digestion with *Nhe I* was expected to produce a 5835‐bp DNA fragment, indicating that the pEMEα vector was not integrated into the host cell's genomic DNA and existed episomally in CHO‐K1 cells.

### Southern analysis

Total DNA isolated from the Hirt extract was digested with *Nhe* I and then subjected to Southern blotting. From pEMEα‐carrying clone #12, 10 and 19, a 5835‐bp restriction fragment identical to the original pEMEα plasmid DNA was obtained (Fig. 4B). The Southern analysis and plasmid rescue experiments further supported the episomal status of plasmid pEMEα in CHO‐K1 cells.

### FISH analysis

**Figure 5:**
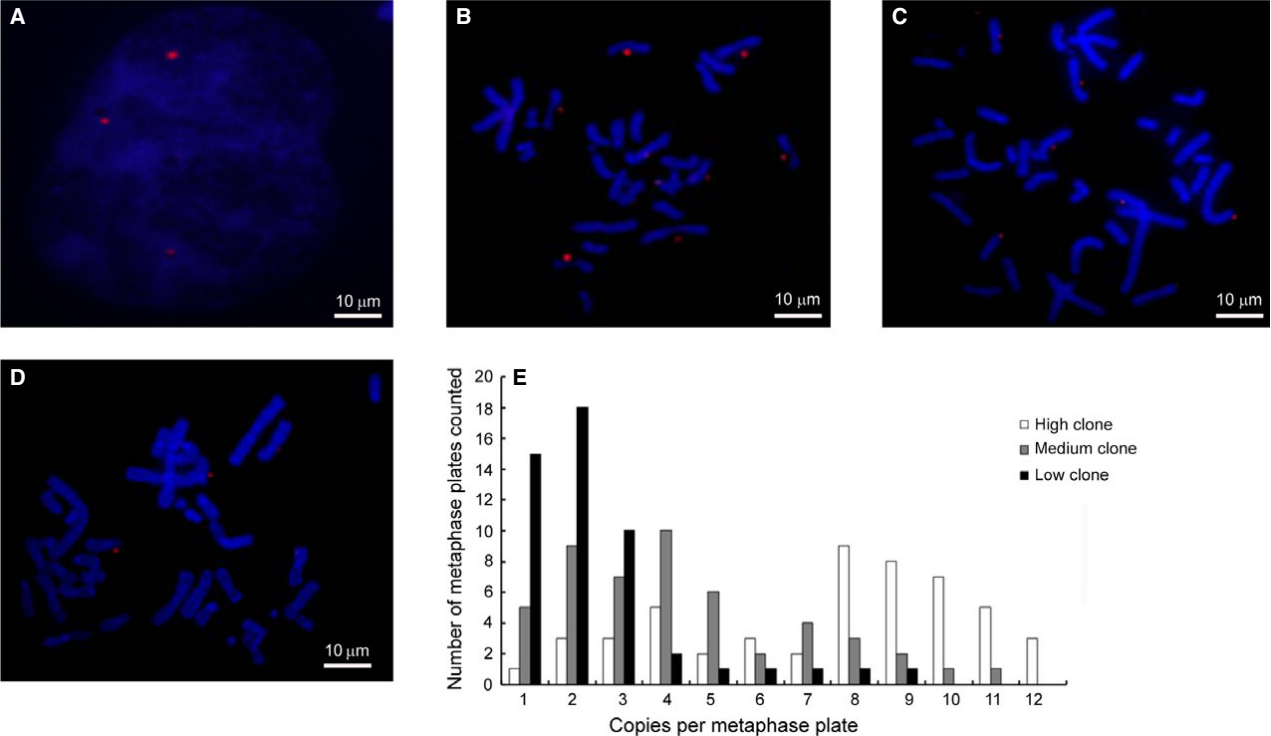
Determination of episomal status and copy numbers with Fluorescence *in situ* hybridization (FISH) (**A**–**D**)CHO‐K1 cells transfected plasmid with EF‐1α were analysed by DNA FISH to assess whether the vectors were present as integrated copies. The episome (red) was visualized by eGFP FISH. Episome localization was determined by FISH on spreads of metaphase chromosomes. (**A**)Vector molecule distribution was monitored in post‐mitotic nuclei of dividing cells; (**B**) clone #12; (**C**) clone #10; (**D**) clone #19. (**E**) Gene copies per metaphase plate as determined by FISH analysis. FISH analysis was performed on monoclonal 12,10,19 cells.

We next evaluated episomal vector replication and analysed copy numbers by FISH analysis, which was performed on spread chromosomes of CHO‐K1 cells transfected with pEMEα (clones #12, 10 and 19). This analysis revealed that the observed mitotic stability of the vector was a result of its episomal presence on metaphase spreads (Fig. 5A–D). Fifty metaphase spreads were analysed by FISH for each clone. An average vector copy number of 7.56 ± 3.18 was estimated in CHO‐K1 cells in the high‐expression group (clone #12; range, 1–12 copies per cell; Fig. 5B, E); in the medium‐expression group, the copy number was 4.37 ± 2.96 (clone #10; range, 1–11 copies per cell; Fig. 5C, E); and in the low‐expression group, the copy number was 2.48 ± 1.03 (clone #19; range, 1–9 copies per cell; Fig. 5D, E).

**Figure 6:**
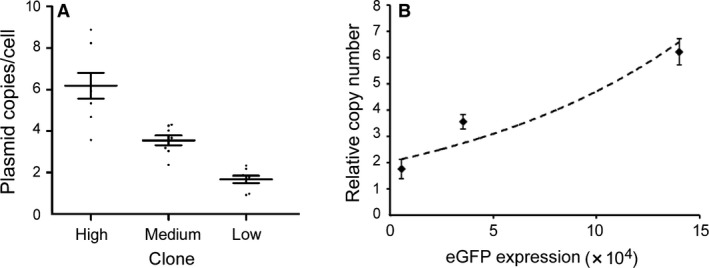
Gene copy numbers in transfected CHO‐K 1 cells with pEMEα. (**A**) The copy number was assessed by quantitative PCR analysis. Clone #12 cells (high clone) established with a copy number of 6.23 ± 1.90, clone #10 cells (medium clone):3.56 ± 1.18 and clone #19 cells (low clone): 1.75 ± 0.86 plasmid copies per cell. (**B**) Correlation between relative copy number and eGFP expression level. Relative copy number analysed by qPCR, generally increased as eGFP expression increased.

### Quantitative PCR

To study the relation between the expression levels and copy number of the episomal vector, the number of plasmid copies per cell for each clone was analysed by quantitative PCR. The high‐expression clone was estimated to have a slightly greater copy number (6.23 ± 1.90), the medium‐expression clone was found to contain 3.56 ± 1.18 copies/cell, while the low‐expression clone showed a slightly lower copy number (1.75 ± 0.86; Fig. 6A). The results suggested that the expression level of eGFP was related to the gene copy number (Fig. 6B).

### Long‐term stability of expression of the recombinant protein

**Figure 7:**
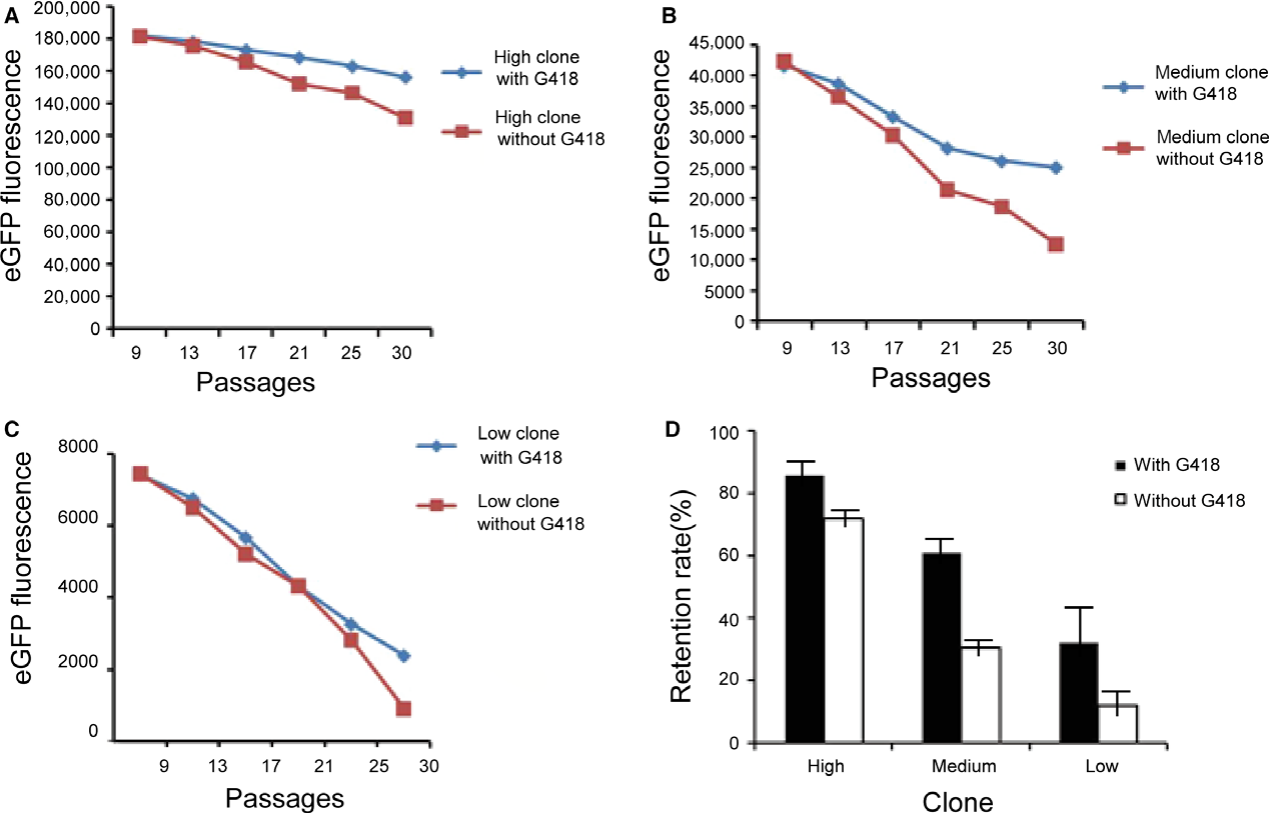
Long‐term transgene expression stability in transfected CHO‐K1 cells. Single‐cell clones of high‐, middle‐ and low‐expression cells for long‐term recombinant protein expression stability analysis. The cells were split in two and further cultured in either the presence or the absence of G418, and the MFI measured to assess the stability of the expressed recombinant protein after 30 generations. Retention rate was calculated according to the eGFP protein levels with or without G418, respectively. Each data point represents mean ± S.D. of triplicate determinations. (**A**) High‐expression monoclonal cells; (**B**) middle‐expression monoclonal cells; (**C**) low‐expression monoclonal cells; (**D**) retention rate.

All stable monoclonal cells were cultured further in either the presence or absence of G418, and MFI was measured in the cells to assess stability of expression of the recombinant protein at passages 9, 13, 17, 21, 25 and 30 post‐transfection. In single‐cell clones, the eGFP levels decreased gradually over time. The eGFP expression in the high‐expression group was higher than that in the medium‐expression and low‐expression groups at passage 30 post‐transfection (Fig. 7A–C). The most stable expression was achieved in the high‐expression group, which retained 86.45% of the original expression level by passage 30 post‐transfection in the presence of G418 selection pressure (Fig. 7D), and retained 72.18% of the original expression level at passage 30 post‐transfection in the absence of G418 selection pressure (Fig. 7D). In contrast, the low‐expression group showed a decrease during the long‐term culture: only 32.37% was retained at passage 30 post‐transfection in the presence of G418 selection pressure and 12.69% in the absence of G418 selection pressure (Fig. 7D).

**Figure 8:**
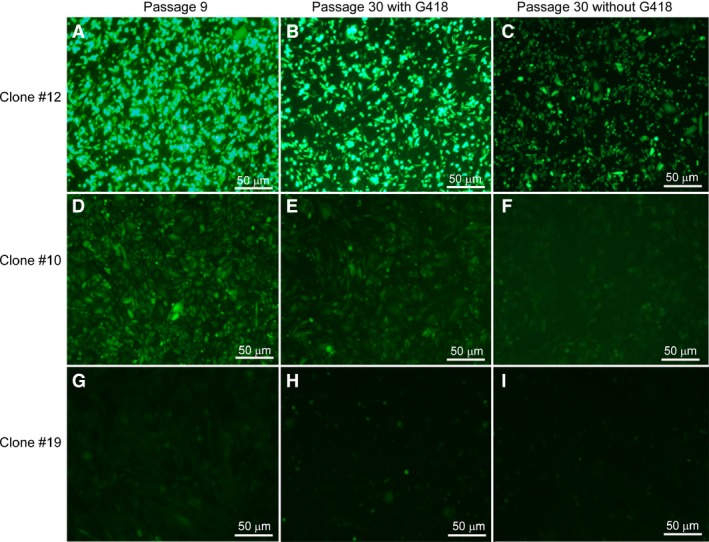
Fluorescence microscopy of the eGFP transfected in CHO K1 cells with the plasmid pEMEα grown in the presence or absence of G418 selection pressure. (**A**,** D**,** G**): Clone #12,10,19 cells cultured for nine generations; (**B**,** E**,** H**): 30 generations in the presence of selection pressure of clone #12,10,19 cells; (**C**,** F**,** I)**: 30 generations in the absence of selection pressure of clone #12,10, 19 cells.

In agreement with the MFI results, eGFP gene expression was also evident in CHO‐K1 by fluorescence microscopy after 30 generations in culture, both under selection pressure and in the absence of selection pressure (Fig. 8).

### Analysis of the effects of transcription factor regulatory elements (TFREs) on transgene expression

Promoter activity is related to transcription factor‐binding sites and TFREs. The distributions of seven TFREs (SP1, NF‐κB, STAT, HSF, GATA, TEF and CEBP) were assessed for the EF‐1α, CAG and CMV promoters. The CEBP, NFκB, STAT, GATA and HSF TFREs were abundant in the EF‐1α promoter (Table 1). The CAG promoter did not contain CEBP and GATA TFREs, and the CMV promoter did not contain GATA TFREs. The findings led us to conclude that TFREs CEBP, GATA and HSF contribute to promoter activity the most.

**Table 1:** Locations of various transcription factor‐binding motifs within the three promoters

Promoter	Strand	AP1	NFkB	STAT	HSF	GATA	TEF	CEBP
CMV	+	4	4	2	2	0	0	0
	−	3	0	1	0	0	1	1
CAG	+	2	5	3	2	0	0	0
	−	3	0	1	0	0	1	0
EF‐1α	+	2	3	3	2	0	2	2
	−	2	2	2	2	2	0	1

## Discussion

Cell lines combining high production and stability are important for recombinant protein production, particularly in relation to proteins for gene therapy. Successful generation of overproducing cell lines requires creation of cell clones expressing the recombinant protein at high and stable magnitude. In this study, we attempted to improve the pEM vector function in terms of transgene expression magnitude, copy number and long‐term stability by inserting three different enhancer elements, mutating the CMV promoter, and using strong promoters CAG and EF‐1α. We found that the use of enhancers, mutating the CMV promoter, and the use of strong promoters increased the transfection efficiency and transient expression of the recombinant protein. In particular, we observed increased long‐term stability and copy number under the influence of the EF‐1α promoter especially in the high‐expression clones .

CMV is prone to transcriptional silencing, which is associated with DNA methylation 27, 28. Mammalian DNA is predominantly methylated at cytosine bases that are part of CpG dinucleotides 29, 30. In the CMV promoter, the cytosines at positions 404 and 542 were found to be methylated frequently 16. To test whether removal of CpG sites can stabilize CMV promoter‐driven gene expression, we point‐mutated C^404^ and C^542^ to G and studied the effect of these mutations on long‐term expression stability in transfected CHO‐K1 cells. The results indicated that CMV promoter mutation increased only the recombinant protein expression transiently and did not affect long‐term stability, which is inconsistent with the results of Benjamin *et al*. 16, who reported that a single mutation of C^‐179^ to G can stabilize the production of a recombinant protein significantly under the control of the CMV promoter in stably transfected CHO cells. This discrepancy may be explained by the fact that Benjamin *et al*. used integrating vectors to construct the CMV promoter mutants, whereas we used an episomal vector. The episomal vector was constructed using a MAR element, and the transgenic expression level was related not only to the promoter but also to other regulatory elements of the vector.

An enhancer is a DNA sequence that can determine the temporal and spatial specificity of expression and increase a promoter's activity. According to one study 31, we synthesized three different enhancer elements and added them (separately) upstream of the promoter. The three different enhancer elements included combinations of NF‐κB, E‐box, GC‐box and C/EBPα elements. The results showed that Enhancer‐1 increased only the recombinant protein expression transiently, without an effect on long‐term stability. Enhancers 2 and 3 did not yield any transcriptional enhancement. An enhancer is a *cis*‐acting element that can increase transcription activity only when combined with tissue‐specific transcription factors and is related to its controlled promoter. Hence, we cannot rule out that the transcription‐promoting effect of an enhancer relies, to some extent, on elements associated with the promoter in question. Transcription factors are different between transient expression and stable expression; and this state of affairs might have resulted in Enhancer‐1′s functioning only in transient expression. In addition, the CMV promoter (589 bp long) contains its own enhancer elements, and a new enhancer upstream of the promoter may not necessarily increase the promoter activity any further.

Transgene expression stability and magnitude are influenced by various components of a plasmid vector, including the promoter. The EF‐1α promoter is known as one of the strongest promoters in various mammalian cell lines 32, and the CAG promoter has been used frequently to drive strong gene expression in mammalian cells. However, in the present study, the CAG promoter could not maintain transgene expression stably during long‐term culture as compared with the CMV promoter. Moreover, the EF‐1α promoter showed the highest promoter activity and tended to be more stable in long‐term culture, even in the absence of selection pressure on the transfected CHO‐K1 cells. The activity of a promoter thus depends on many factors, such as genomic *cis*‐acting sequences, cell line, type of vector and transcription factor‐binding sites. Yang *et al*. demonstrated that the CAG promoter can drive transgene expression in chick embryo cells 33, whereas we transfected CHO cells. This difference may explain the weaker expression of the transgene under the control of the CAG promoter in transfected CHO cells. As mentioned above, promoter activity is affected by transcription factor‐binding sites or TFREs. The TFREs of the promoters used in this study were analysed, and the results revealed that the CEBP and GATA TFREs are abundant in the EF‐1α promoter, but GATA is absent in the CMV and CAG promoters. These findings lead us to hypothesize that in episomal vectors, the CEBP and GATA TFREs contribute to the promoter activity the most.

Our results revealed that the EF‐1α promoter is a potent regulatory sequence for episomal vectors and maintains high transgene expression. Plasmids containing the EF‐1α promoter were found to replicate efficiently, stably and extrachromosomally in CHO‐K1 cells. In addition, we found that the transgene expression magnitude of cells transfected with the EF‐1α promoter‐carrying vector is associated with the copy number: an elevated copy number caused higher expression.

In this study, we investigated the activity of various promoters and promoter‐variant vectors in transfected CHO‐ K1 cells. However, only the CHO‐K1 cell line was tested here, and the vectors should be evaluated in other cell lines and *in vivo*. In addition, other *cis*‐acting elements in expression vectors need to be optimized further to develop a high‐efficiency expression system for gene therapy.

## Conflict of interest

All authors have no conflict of interest regarding this paper.

## Supporting information


**Figure S1** Schematic illustration of expression vectors containing different element.
**Figure S2** Promoter and Enhancer sequences used in this study.
