# Supplementary material for: The EF‐1α promoter maintains high‐level transgene expression from episomal vectors in transfected CHO‐K1 cells
Source: J Cell Mol Med. 2017 May 30;21(11):3044–54. doi: 10.1111/jcmm.13216 (PMC5661254; doi:10.1111/jcmm.13216)
Supplement: Supplementary file 1 — Figure S1 Schematic illustration of expression vectors containing different element. Figure S2 Promoter and Enhancer sequences used in this study. [file JCMM-21-3044-s001.docx]

**The EF-1α promoter maintains high-level transgene expression from episomal vectors in transfected CHO-K1 cells**

**Xiaoyin Wang^a,b,#^, Chongjie Xu^c,#^, Zhengwei Tian^a,#^, Qin Li^d^, Junhe Zhang^a^,Tianyun Wang^a*^**

*^a^Department of Biochemistry and Molecular Biology, Xinxiang Medical University, Xinxiang 453003, Henan, China*

*^b^Henan Collaborative Innovation Center of Molecular Diagnosis and Laboratory Medicine, Xinxiang 453003, Henan, China*

*^c^Life Science and Technology, Xinxiang Medical University, Xinxiang 453003, Henan, China*

*^d^Test Laboratory,* *Xinxiang Medical University, Xinxiang 453003, Henan, China*

**Fig. S1 Schematic illustration of expression vectors containing different element.**

All vectors were derived from the pEM (**A**),which comprise a characteristic MAR motif from the human IFN-β gene was cloned into the multiple cloning site (MCS) of pEGFP-C1 resulting in the vector pEM.

(**B**) The CMV promoter mutants: the cytosine at positions 404 and 542 in the CMV promoter seem to be frequently methylated, so we point-mutated C 404, C542 to G to create two CMV mutants episomal vectors respectively, pEMMu1 and pEMMu2 .

(**C**) Three different enhancer elements added upstream of the CMV promoters to create three episomal vectors with enhancer respectively, pEMEn1, pEMEn2,and pEMEn3.

(**D-E**) pEMEα and pEMC were generated by CMV promoter was excised and replaced by EF-1α and CAG promoters.

**
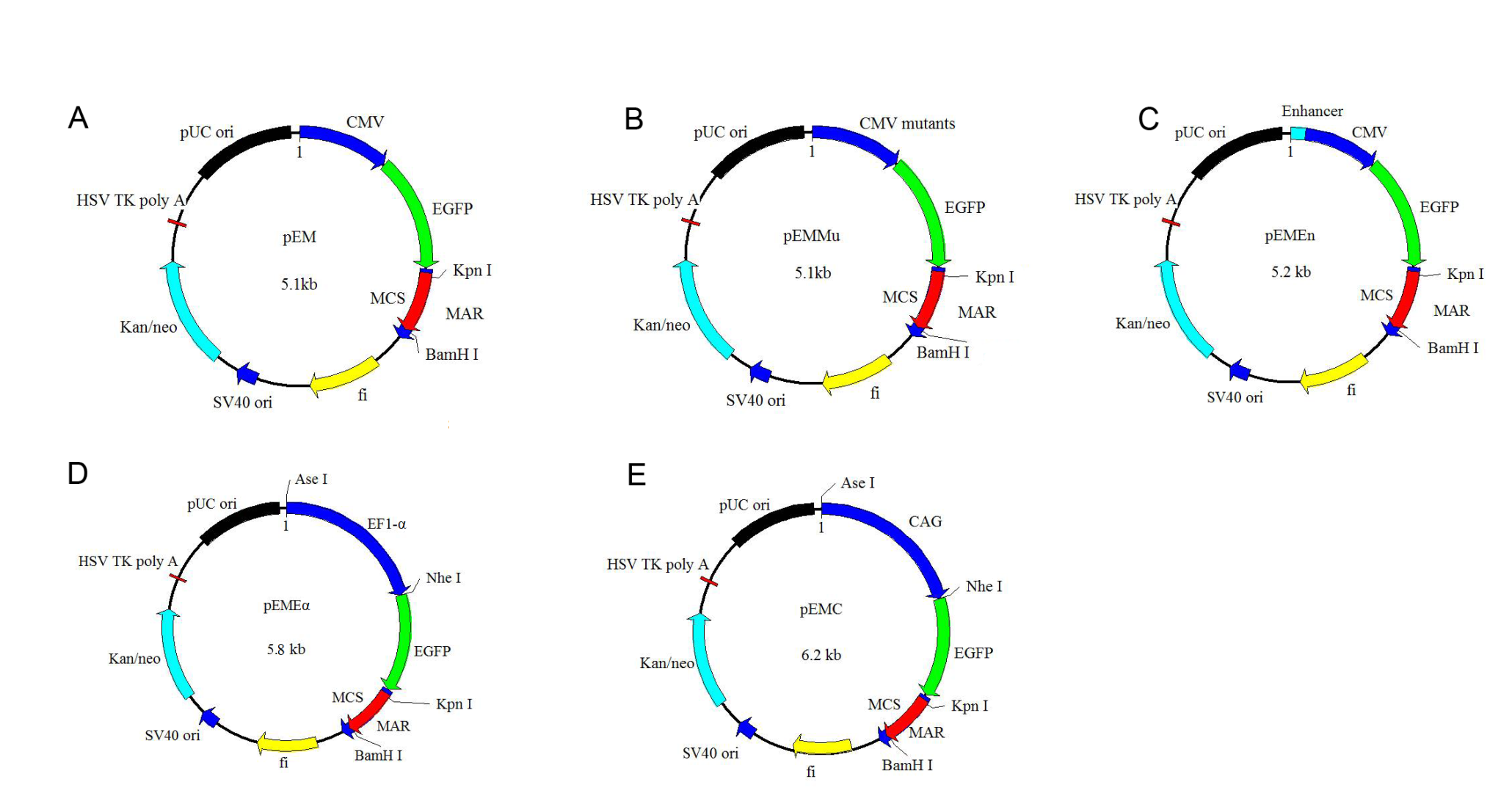
**

**Figure S2 Promoter and Enhancer sequences used in this study**

**CAG promoter sequence (1662bp)**

Tagttattaatagtaatcaattacggggtcattagttcatagcccatatatggagttccgcgttacataacttacggtaaatggcccgcctggctgaccgcccaacgacccccgcccattgacgtcaataatgacgtatgttcccatagtaacgccaatagggactttccattgacgtcaatgggtggagtatttacggtaaactgcccacttggcagtacatcaagtgtatcatatgccaagtacgccccctattgacgtcaatgacggtaaatggcccgcctggcattatgcccagtacatgaccttatgggactttcctacttggcagtacatctacgtattagtcatcgctattaccatggtcgaggtgagccccacgttctgcttcactctccccatctcccccccctccccacccccaattttgtatttatttattttttaattattttgtgcagcgatgggggcggggggggggggggggcgcgcgccaggcggggcggggcggggcgaggggcggggcggggcgaggcggagaggtgcggcggcagccaatcagagcggcgcgctccgaaagtttccttttatggcgaggcggcggcggcggcggccctataaaaagcgaagcgcgcggcgggcggggagtcgctgcgacgctgccttcgccccgtgccccgctccgccgccgcctcgcgccgcccgccccggctctgactgaccgcgttactcccacaggtgagcgggcgggacggcccttctcctccgggctgtaattagcgcttggtttaatgacggcttgtttcttttctgtggctgcgtgaaagccttgaggggctccgggagggccctttgtgcggggggagcggctcggggggtgcgtgcgtgtgtgtgtgcgtggggagcgccgcgtgcggctccgcgctgcccggcggctgtgagcgctgcgggcgcggcgcggggctttgtgcgctccgcagtgtgcgcgaggggagcgcggccgggggcggtgccccgcggtgcggggggggctgcgaggggaacaaaggctgcgtgcggggtgtgtgcgtgggggggtgagcagggggtgtgggcgcgtcggtcgggctgcaaccccccctgcacccccctccccgagttgctgagcacggcccggcttcgggtgcggggctccgtacggggcgtggcgcggggctcgccgtgccgggcggggggtggcggcaggtgggggtgccgggcggggcggggccgcctcgggccggggagggctcgggggaggggcgcggcggcccccggagcgccggcggctgtcgaggcgcggcgagccgcagccattgccttttatggtaatcgtgcgagagggcgcagggacttcctttgtcccaaatctgtgcggagccgaaatctgggaggcgccgccgcaccccctctagcgggcgcggggcgaagcggtgcggcgccggcaggaaggaaatgggcggggagggccttcgtgcgtcgccgcgccgccgtccccttctccctctccagcctcggggctgtccgcggggggacggctgccttcgggggggacggggcagggcggggttcggcttctggcgtgtgaccggcggctctagagcctctgctaaccatgttcatgccttcttctttttcctacag

**EF-1α promoter sequence (1335bp)**

GAGTAATTCATACAAAAGGACTCGCCCCTGCCTTGGGGAATCCCAGGGACCGTCGTTAAACTCCCACTAACGTAGAACCCAGAGATCGCTGCGTTCCCGCCCCCTCACCCGCCCGCTCTCGTCATCACTGAGGTGGAGAAGAGCATGCGTGAGGCTCCGGTGCCCGTCAGTGGGCAGAGCGCACATCGCCCACAGTCCCCGAGAAGTTGGGGGGAGGGGTCGGCAATTGAACCGGTGCCTAGAGAAGGTGGCGCGGGGTAAACTGGGAAAGTGATGTCGTGTACTGGCTCCGCCTTTTTCCCGAGGGTGGGGGAGAACCGTATATAAGTGCAGTAGTCGCCGTGAACGTTCTTTTTCGCAACGGGTTTGCCGCCAGAACACAGGTAAGTGCCGTGTGTGGTTCCCGCGGGCCTGGCCTCTTTACGGGTTATGGCCCTTGCGTGCCTTGAATTACTTCCACGCCCCTGGCTGCAGTACGTGATTCTTGATCCCGAGCTTCGGGTTGGAAGTGGGTGGGAGAGTTCGAGGCCTTGCGCTTAAGGAGCCCCTTCGCCTCGTGCTTGAGTTGAGGCCTGGCTTGGGCGCTGGGGCCGCCGCGTGCGAATCTGGTGGCACCTTCGCGCCTGTCTCGCTGCTTTCGATAAGTCTCTAGCCATTTAAAATTTTTGATGACCTGCTGCGACGCTTTTTTTCTGGCAAGATAGTCTTGTAAATGCGGGCCAAGATCTGCACACTGGTATTTCGGTTTTTGGGGCCGCGGGCGGCGACGGGGCCCGTGCGTCCCAGCGCACATGTTCGGCGAGGCGGGGCCTGCGAGCGCGGCCACCGAGAATCGGACGGGGGTAGTCTCAAGCTGGCCGGCCTGCTCTGGTGCCTGGCCTCGCGCCGCCGTGTATCGCCCCGCCCTGGGCGGCAAGGCTGGCCCGGTCGGCACCAGTTGCGTGAGCGGAAAGATGGCCGCTTCCCGGCCCTGCTGCAGGGAGCTCAAAATGGAGGACGCGGCGCTCGGGAGAGCGGGCGGGTGAGTCACCCACACAAAGGAAAAGGGCCTTTCCGTCCTCAGCCGTCGCTTCATGTGACTCCACGGAGTACCGGGCGCCGTCCAGGCACCTCGATTAGTTCTCGAGCTTTTGGAGTACGTCGTCTTTAGGTTGGGGGGAGGGGTTTTATGCGATGGAGTTTCCCCACACTGAGTGGGTGGAGACTGAAGTTAGGCCAGCTTGGCACTTGATGTAATTCTCCTTGGAATTTGCCCTTTTTGAGTTTGGATCTTGGTTCATTCTCAAGCCTCAGACAGTGGTTCAAAGTTTTTTTCTTCCATTTCAGGTGTCGTGA

**Enhancer 1 sequence (111 bp)**

GGGACTTTCCGGGGCGGGGCACGTGGTGCACGGGACTTTCCGTGCACGTGCACGGGACTTTCCGGGACTTTCCGGGACTTTCCGTGCACCACGTGGGGACTTTCCGTGCAC

**Enhancer 2 sequence (117 bp)**

CACGTGCACGTGCCTTTCAGGGGGGGCGGGGCACGTGCCTTTCAGGGGTGCACCCTTTCAGGGCCTTTCAGGGGTGCACCCTTTCAGGGCCTTTCAGGGGGGACTTTCCTTGCGCAA

**Enhancer 3 sequence (113 bp)**

CCTTTCAGGGGGGACTTTCCGGGGCGGGGGGGACTTTCCAACGCGTTGTGCACGTGCACGGGACTTTCCTTGCGCAAGGGACTTTCCGTGCACGGGACTTTCCCCTTTCAGGG
